# Pharmacy-based immunization: a systematic review

**DOI:** 10.3389/fpubh.2023.1152556

**Published:** 2023-04-13

**Authors:** Marisol S. Romero-Mancilla, Jaime Mora-Vargas, Angel Ruiz

**Affiliations:** ^1^Tecnologico de Monterrey, School of Engineering and Science, Monterrey, Mexico; ^2^Faculty of Business Administration, Laval University, Quebec, QC, Canada

**Keywords:** health system, pharmacy-based immunization, humanitarian supply chain management, humanitarian logistics, immunization, vaccination

## Abstract

**Background:**

The coronavirus disease 2019 pandemic has prompted the exploration of new response strategies for such health contingencies in the near future. Over the last 15 years, several pharmacy-based immunization (PBI) strategies have emerged seeking to exploit the potential of pharmacies as immunization, medication sale, and rapid test centers. However, the participation of pharmacies during the last pandemic was very uneven from one country to another, suggesting a lack of consensus on the definition of their roles and gaps between the literature and practice.

**Purpose:**

This study aimed to consolidate the current state of the literature on PBI, document its progress over time, and identify the gaps not yet addressed. Moreover, this study seeks to (i) provide new researchers with an overview of the studies on PBI and (ii) to inform both public health and private organization managers on the range of possible immunization models and strategies.

**Methodology:**

A systematic review of scientific qualitative and quantitative studies on the most important scientific databases was conducted. The Preferred Reporting Items for Systematic Reviews and Meta-analyzes guidelines were followed. Finally, this study discusses the trends, challenges, and limitations on the existing literature on PBI.

**Findings:**

Must studies concluded that PBI is a beneficial strategy for the population, particularly in terms of accessibility and territorial equity. However, the effectiveness of PBI is affected by the economic, political, and/or social context of the region. The collaboration between the public (government and health departments) and private (various pharmacy chains) sectors contributes to PBI's success.

**Originality:**

Unlike previous literature reviews on PBI that compiled qualitative and statistical studies, this study reviewed studies proposing mathematical optimization methods to approach PBI.

## 1. Introduction

Infectious diseases outbreaks such as the black death (1347), the smallpox (1520), cholera (1817), or the Spanish flu (1918), resulted in millions of deaths, increased calamities, collapsed health services, economic crises, and political conflicts (1). The increase in mobility of people between cities (2), the interaction between humans, animals, and ecosystems (3), and the transmission of zoonotic pathogens from animals to humans, have potentiated the emergence and recurrence of epidemics and pandemics, which have caused a millions of deaths. Fortunately, due to medical, scientific, and technological advances to mitigate the impact of pandemics, the mortality rates attributed to them, have decreased (3).

To cope with infectious diseases' outbreaks, strategies have been developed through history to reduce their transmission and mortality. Some of them include the practice of *quarantine* which arose during the Black death (4), *vaccination and collective immunity, contact tracking* emerged during syphilis (1930s) to slow down the propagation of it, and *social distancing* with the Influenza H1N1 (2009) pandemic. Among the former, mass vaccination has proved so far as the most efficient strategy to cope with infectious diseases' outbreaks. Indeed, mass vaccination has made possible to stop the spread of infectious diseases, prevent the resurgence of known and treatable diseases (3), eradicate some diseases such as smallpox, and to reduce significantly the incidence of other infectious diseases such as polio and diphtheria, tetanus, measles, and meningitis, among others (5, 6).

Unfortunately, it is expected that the emergence and spread of infectious diseases will increase, and with them, the need for more resilient health systems able to anticipate, prepare, and respond more efficiently to pandemics. In this vein, governments and international agencies must lead the design, and implementation of response strategies (7), and ensure the control of the flow and storage of supplies and services to adequately protect the populations.

In this context, this paper focuses on the literature studying the role of pharmacies, alone or in collaboration with other public or private immunization facilities, in mass vaccination networks. This vaccination modality is referred to as Pharmacy-Based Immunization (PBI). Intuitively, pharmacies can play a key role in vaccination networks to balance their global efficiency and accessibility. On the one hand, mass vaccination centers are very effective and efficient. On the other hand, they are expensive and require a high flow of patients, so their number must be constrained and individuals from some regions must travel long distances to get the service. Pharmacies are considered first-contact care points due to their proximity to the final patient, they spread over the territory, thus guarantying the population a facile access to vaccines. Moreover, they employ professionals that are (or can easily) trained to handle vaccination activities, including the required logistics and public management tasks, such as appointments scheduling.

Although PBI strategies have been successfully implemented in some countries, the recent COVID-19 pandemic shown that other countries simply neglected PBI. We therefore aim to review and consolidate the available literature on PBI in order to better assess their potential application while discussing their limitations from an operations research perspective. It is important to highlight that in a PBI strategy the pharmacy chains are owned and managed by the private sector (individuals or corporations), and the government of each country agrees with them performing immunization activities. Therefore, this research is developed under those conditions and the research questions that arose are:

RQ1. *What are the reported results of PBI implementation?*
RQ2. *Where and how PBI has been executed?*
RQ3. *What should be necessary (from research) to support a better implementation of PBI strategies?*

The objective of this review is to contribute with the answers to these questions as well as with an analysis of the current state of the literature on PBI, its progress over time, and the gaps that have not been yet addressed. Moreover, this research is directed to the general public and the scientific community, but may be of greater interest to those in charge of managing immunization campaigns in the health sector, and managers from private sectors that provide health services. Either way, this research can guide them to complement, adapt, develop, implement and/or inquire into PBI strategies.

The relevance of this study lies in the importance of studying immunization as a global public health management issue to broaden the range of possible vaccination strategies that can be implemented in various countries that have not yet tried this strategy because they do not know it or because they are still reluctant to use it due to lack of information. In the scientific and public health management field, it is useful to show the limitations that exist in the subject and the information gaps that can give rise to future research.

The paper is structured as follows. In Section 2 the methodology to develop this paper is described. Section 3 discusses the literature review on PBI, Section 2.6 shows the results of this study, and in Section 4, the discussion is presented. Finally, in Section 5 some conclusions and suggestions for further research are outlined.

## 2. Methodology

**Figure 1:**
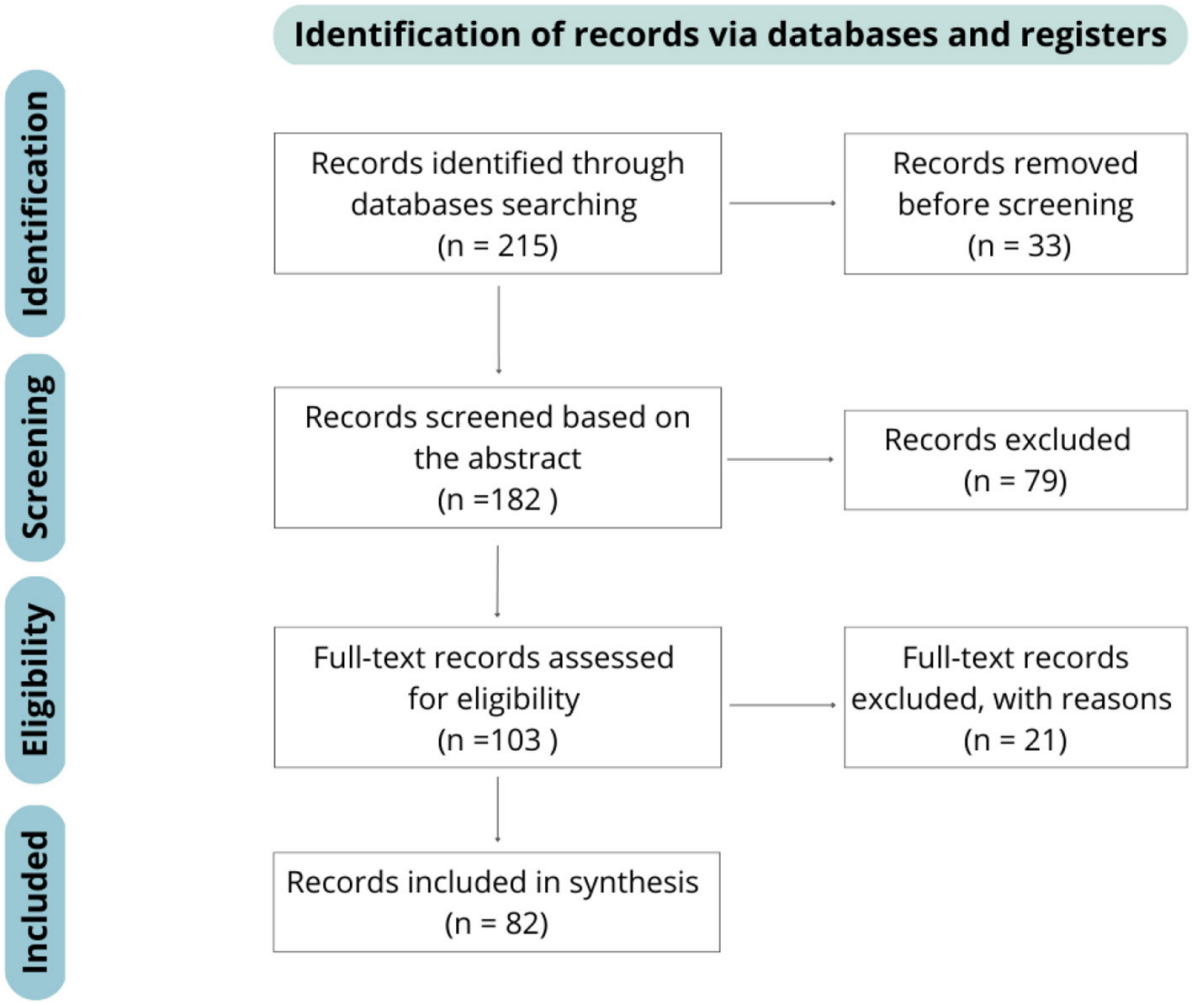
PRISMA flow diagram.

The Preferred Reporting Items for Systematic Reviews and Meta-analyzes (PRISMA) guidelines were followed for the analysis of the literature. PRISMA is a methodology developed for doing systematic reviews and meta-analyzes. It consists of an evidence-based minimum set of items for a better analysis, and a transparent reporting of the review's objective, what was done, and what was found (8). Figure 1 summarizes the PRISMA flow diagram and its application to our research.

### 2.1. Elegibility criteria

Publications in English and Spanish were reviewed. Moreover, quantitative, and qualitative studies such as academic publications (journal articles, proceedings and theses) ranging from the oldest found to the latest published in mid-2022, were included. Lastly, the publications (9–11) were suggested by the reviewers during the interactive reviewing process.

### 2.2. Information sources

To select the articles presented in this survey, Google, Google Scholar, Scopus, and ResearchGate were used as the principal search engines. Other bibliographic databases consulted were PubMed, Web of Science, Springer, and Science Direct.

### 2.3. Search strategy

To initialize the publications search with Google Scholar, Scopus, and ResearchGate, 4 groups of keywords were used with different combinations among each group.

- Group 1: “pharmacy” and “rural” and “vaccination”
- Group 2: “pharmacy” and {“immunization” or “vaccination”} and {“Malaria” or “Influenza” or “antiretroviral”} and {“optimization” or “facility location” or “coverage”}
- Group 3: “geospatial” and pharmacy” and {“immunization” or “vaccination”} and {“optimization” or “facility location” or “coverage”}
- Group 4: {“pharmacy” or “pharmacy-based”} and {“vaccine” or “immunization”} and {“optimization” or “facility location” or “coverage”}.

For Web of Science, PubMed, Springer and Science Direct only one group of words was used: “Pharmacy-Based Immunization” since trying to add more keywords like “optimization,” complicated the search and no more results came out or the results did not align with the topic of this research.

It is worth noting that when using the keywords “pharmacy” and “immunization” to look for the papers for this research, the results yielded many studies that were related to the role of pharmacists in encouraging vaccination among the population and studies that advocated allowing pharmacists vaccinate populations, representing a bottleneck in finding the appropriate papers for this research as they were not 100% compatible with what was sought. On the other hand, by adding the keyword “optimization” the results were reduced in Google Scholar and in databases such as Web of Science no results were shown.

### 2.4. Selection process

In the first screening, the resulting list of papers was analyzed according to their titles to eliminate duplicates. A second screening was executed, in which all the abstracts of the list were analyzed and the articles that did not contribute to answer the research questions were eliminated. Finally, the full content of the remaining articles was analyzed to decide if they were eligible to be included in the review. Articles presenting different mathematical approaches for immunization problems were included if they were useful to deal with PBI problems.

### 2.5. Data collection and extraction

**Figure 2:**
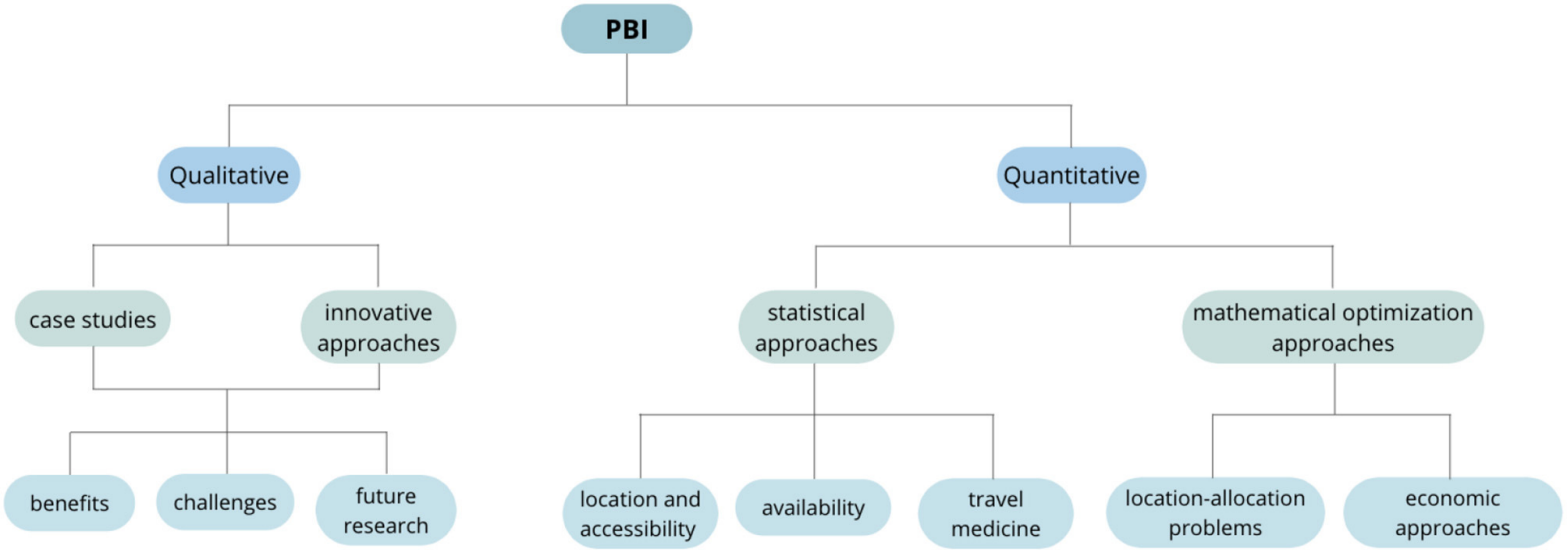
Grouping scheme of papers related to pharmacy-based immunization.

**Figure 3:**
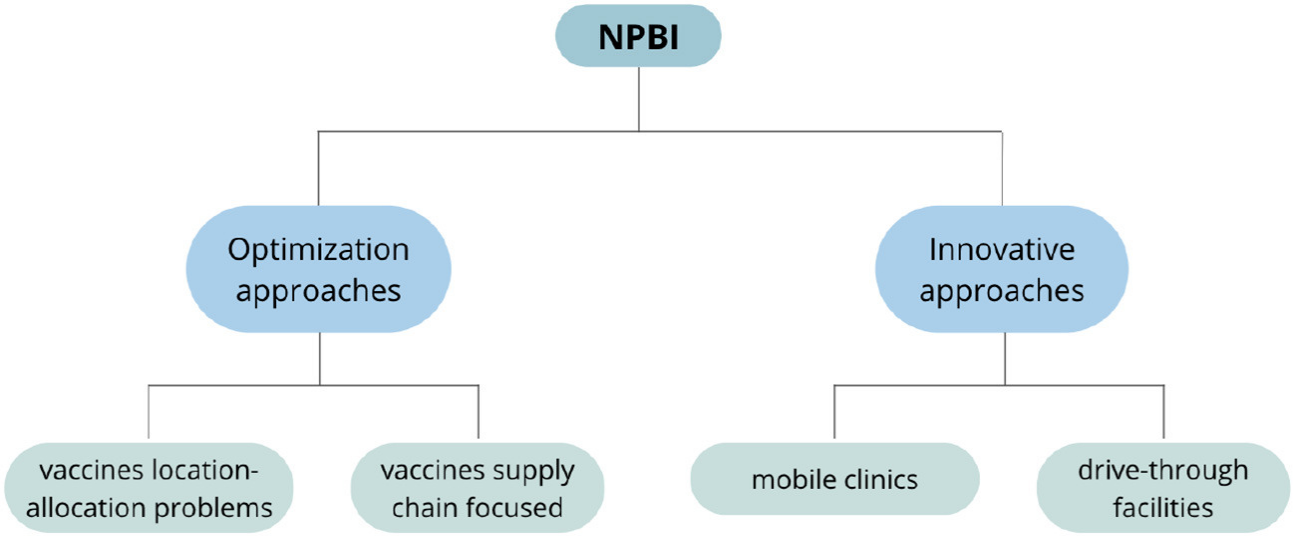
Grouping scheme of non-pharmacy-based immunization related papers.

The articles were grouped depending on the type of study they carry out into qualitative and quantitative. A third category called Non-Pharmacy-Based Immunization (NPBI) approaches was created to group studies that, although do not explicitly considers pharmacies, can be adapted and used to improve PBI strategies. The qualitative articles were used to introduce and contextualize the topic and to identify opportunity areas on PBI; they consist of surveys, reviews, interviews, and case studies. Quantitative articles were classified into statistical and optimization approaches. The statistical approaches include interviews, surveys, cross-sectional studies, case studies, and statistical analyzes; optimization approaches include case studies, location and allocation problems, heuristics, and exact methods. The classification scheme is illustrated in Figures 2, 3.

Specific information was extracted from each type of paper to fill Tables 2, 3, 4 in Appendix. The qualitative studies were carefully read, and key aspects were identified, such as benefits, limitations, and opportunities of implementing PBI strategies. Among the quantitative studies, statistical approaches were used mainly to obtain insights on previous applications of PBI strategies, the opinion of medical staff and patients on the application of this type of strategies, and improvement opportunities. While the optimization-focused studies were used to compare methodologies used to solve location and allocation problems in PBI strategies and their different objectives. Finally, for all articles, year of publication, country of origin and in case studies, the country where it was applied, were identified.

### 2.6. Result

**Figure 4:**
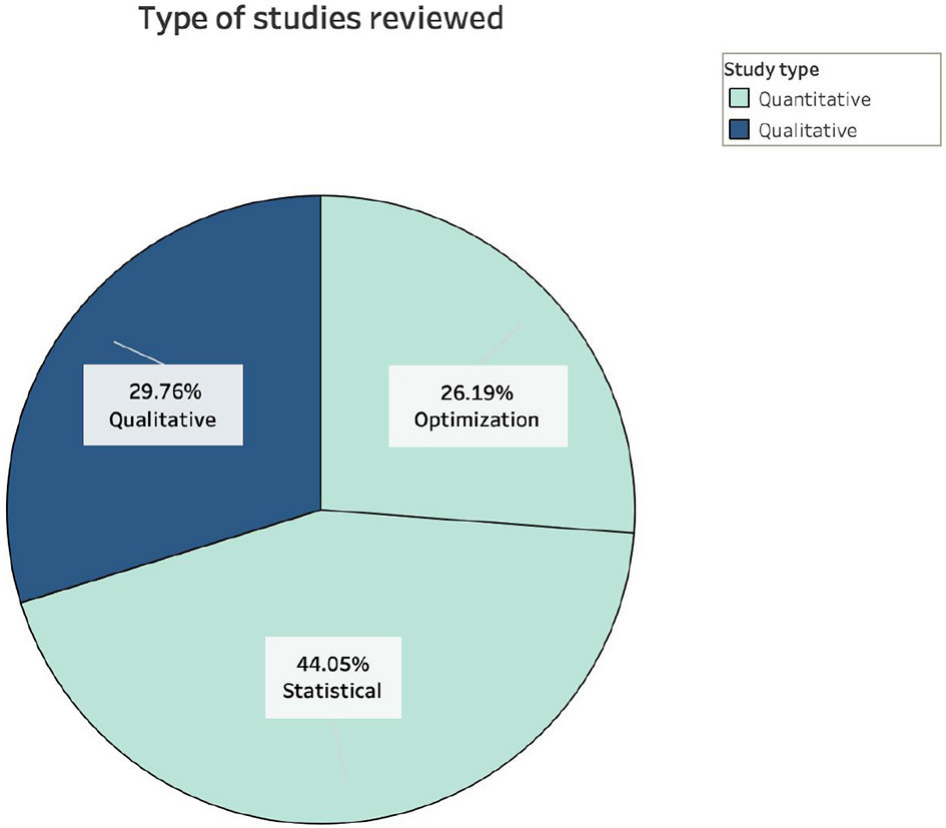
Research in vaccination strategies using PBI for different diseases.

The analysis of the data collected allowed to sketch a first portrait of the research devoted to PBI so far. Firstly, 30% of the papers were classified as qualitative and 70% quantitative. The qualitative studies consisted of reviews, summaries, and case studies, and the quantitative studies consisted of 26% optimization and 44% statistical approaches of the overall studies (Figure 4). Although the proportion of qualitative studies reviewed is lower than that of quantitative ones, an abundance of this type of study was identified in the literature. In addition, many studies with a statistical approach were also identified, including database analyzes, telephone and in-person interviews and questionnaires, case studies, experiments, and cluster-randomized trials. Paradoxically, although the benefits of PBI strategies and the importance of locating facilities in the immunization supply chain to maximize coverage and reduce costs are known, only a small number of studies with an optimization approach for the location of pharmacies was found, therefore research focused on the location of facilities for vaccination and testing strategies were included as well, even if they were not focused on the use of pharmacies.

**Figure 5:**
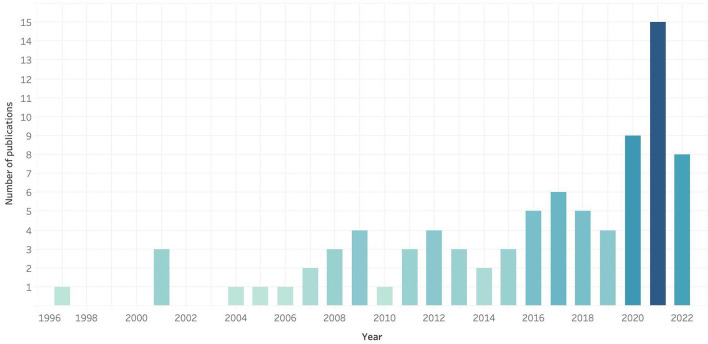
Number of publications related to PBI per year.

**Figure 6:**
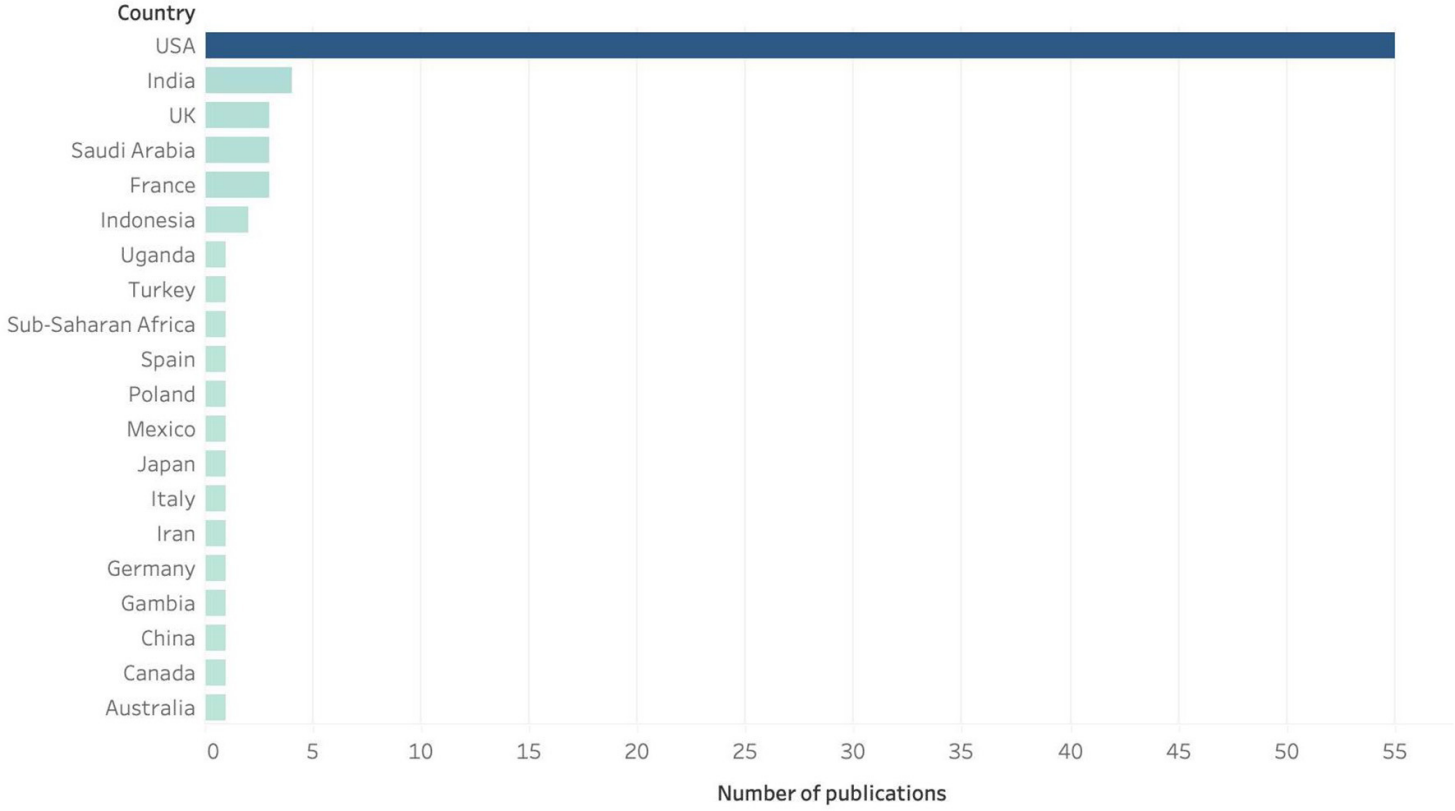
Publications per country.

If we look at the number of papers published yearly, we observe a continuous increase with marked leaps in years 2001, 2008–2009, 2012, 2016, 2017, and 2021, as shown in Figure 5. As per the geographic distribution of the publications, is noticeable that most of the research about of PBI has been published by researchers in the USA, followed by India, UK, Saudi Arabia and France (see Figure 6); the country on which most of the PBI case studies were carried out are, again, the United Stated, where several PBI strategies have been already implemented in various states, followed by India and Indonesia. Although it is known that several European countries use this strategy to immunize their population, there are not so many publications that can be found on this subject; in Canada, there are more studies related to the use of pharmacists as immunizers than PBI strategies.

## 3. Literature review: Pharmacy-Based Immunization

This section seeks to better position the research on PBI by analyzing the contributions of the reviewed studies. The section is divided into four parts. The first part aims to clarify the role of pharmacies providing basic, first-contact care. Then, in the second and third parts, the qualitative and quantitative contributions in which pharmacies are used as vaccination centers to immunize a population are reviewed. The fourth and last part reviews and analyzes non-PBI (NPBI) studies.

### 3.1. First-contact care

Most healthcare systems structure their service offering on a hierarchical scheme dividing care into primary, secondary and tertiary, depending on their level of specialization (12). Primary or first-contact care is defined as the use of an identified primary care resource for the first medical attention visit where patients receive less resource-intensive medical care that do not require the more expensive and specific facilities or skills of hospitals or advanced clinics (13). Once the health problem is identified, the patient's health needs are matched with the appropriate health care resources (13). This health care system can encourage health models move from individual-based medical care that repairs damage, to a model of population-based health care that anticipates risks (14).

Advantages of first-contact care include lower out-of-pocket payments, better accessibility that primary health care institutions (12), proximity to home or workplace (15, 16), short waiting time, good doctor-patient communication, and practitioners' friendly or sincere attitudes (15). Furthermore, Pandhi et al. (17) concluded that first-contact care access for patients who already had a primary care physician is associated with higher receipt of preventive services when compared with having continuity of care alone. From a systematic standpoint, the most relevant benefit of first-contact care is a significant reduction of expenditures that in some case may reach up to 50% (13).

On the other hand, one of the principal problems that face first-contact care facilities promotion is the lack of confidence and/or habit of patients to attend these sites (13). Furthermore, availability of diagnostic equipment, perceived “recovery” (15, 16), the idea that medications provided in these sites are of inferior quality than those in hospitals, and the perception of fist-contact care as a source for follow-ups rather than for effective medical assistance, are other limitations that faces its use (15).

Despite of first-contact's strengths, better accessibility strategies are crucial to improve its effectiveness in practice (12, 18), particularly with respect to the identification and visibility of first-contact points and the care they offer, and its accessibility. In this last direction, Gautham et al. (16) suggests more mobile, virtually doorstep-primary curative care combining consultation and dispensing of medicines within longer business hours. Frenk et al. (14) reports the Mexican “advanced primary health care centers” which offer an enhanced service mix for low-risk deliveries, ambulatory surgery, and first-contact emergency care, these facilities are similar to the US Primary & Urgent Care units.

### 3.2. PBI strategies: Qualitative approaches

PBI has its origins in the United States after the initial influenza vaccination season in 1999, when an expanded protocol was officially approved for all pharmacies to include the administration of tetanus and diphtheria, hepatitis A, hepatitis B, Lyme disease, meningococcal, varicella, and measles, mumps, and rubella vaccines to adults (19). In Great Britain, since 2002, pharmacists have been authorized to deliver flu vaccines privately or use selected patient group directions. In fact, in certain areas of London, 11% of influenza immunizations were performed in rural areas, which represented 30% of all immunizations in individuals older than 65 years. In 2007, Portugal accredited community pharmacists as vaccines agents (5) and implemented PBI with community pharmacies; 13% of the individuals vaccinated in the pharmacies had never got a flu vaccination before. Approximately 36% of all vaccinations were administered in pharmacies (20). In 2012, Ontario, Canada, began an immunization strategy delivered by pharmacists. Western Australia was not left behind and began immunization programs with pharmacists in 2015. In 2017, France provided vaccinations in community pharmacies to increase coverage, and in the same year, Catalonia, Spain, created a collaborative framework to define pharmaceutical care services, which included the application of a vaccine policy (5, 21). Finally, in 2018, Norway revised its national vaccination program to include pharmacies as vaccination centers. Currently, PBI is effectively performed in 13 countries of Europe (22).

### 3.3. Case studies

To date, in the United States several community pharmacies (23) and pharmacy chains (19) have participated to immunization campaigns as immunizers, educators, and facilitators (23). In 1998, the pharmacies affiliated to the American supermarket chain Ukrop participated in an adult immunization program where pharmacists offered influenza and pneumococcal immunizations and diabetes mellitus, hypertension, and hyperlipidemia screenings. The program improved the care level offered to patients and the vaccination rate for adults and simultaneously increased the involvement and enthusiasm to enhance the services (19, 24). The Walgreens^®^ community pharmacies conducted a study (25) on vaccinations administered during off-clinic hours (i.e., evening, weekends, and holidays). The study demonstrated an increase in the number of working-aged healthy adults who accessed the pharmacies to receive the vaccines during off-clinic hours. Nowadays, the US federal government, through the Federal Retail Pharmacy Program (FRPP) for COVID-19 Vaccination, provides COVID-19 vaccines to the American public free of charge collaborating with public health agencies (CDC, state, local, and territorial health departments) (26).

### 3.4. Innovative approaches

Furthermore, innovative approaches that can be complementary to PBI have emerged. The next paragraphs report those that were deemed the most relevant for this research.

The Clinical Pharmacy International Travel Clinic (CPITC) (27) is a telepharmacy service run in the Colorado Region, US, by the Kaiser Permanente nonprofit care and coverage organization, which provides vaccination and health assessment services to travelers. The staff consists of an infectious disease physician, an infectious disease clinical pharmacy specialist, four clinical pharmacists, and a pharmacy technician. The CPITC provides 10-30-min consultations *via* telephone provided by clinical pharmacists; no appointments are required; the patients receive the consultation at the time they call; and only health recommendations regarding traveling are provided. For vaccination services, patients should do a reservation 2 months in advance to let the service plan the number of vaccines or products needed; therefore, only a few patients without a reservation are attended.

An innovative immunization strategy with the collaboration of pharmacy and nursing students in local neighborhood clinics to facilitate access to immunization for the elderly has been reported by Evenson et al. (28). In this case study, the influenza vaccination clinics in Sioux Falls, South Dakota, in addition to providing walk-in services, organized trucks to transport groups of old adults to clinics and immunize as many individuals as possible.

In the US, Walgreens community pharmacies have come with new disease management services through its collaboration with Labcorp, a company specializing in medical biology analyzes. This service includes patient assessment and tests. After that, care is provided. Specifically, Labcorp's staff collects specimens, transports them to the pharmacies, and send them to a central laboratory to process them (29).

Hendricks Pharmacy, a community pharmacy in Claremont, California, offers home delivery, compounding, and blood glucose, blood pressure, and cholesterol screenings. It encourages the use of comprehensive pharmacist-run travel health clinics and strong collaborations among the travel clinic pharmacists, the pharmacy store owners, and physician supervisor for its successful implementation (30).

A prototype mobile app developed by researchers in the UK to verify instant tamper-proof coronavirus disease 2019 (COVID-19) test results to avoid contact with individuals carrying the virus as much as possible between individuals is described in Eisenstadt et al. (31).

In a study in Bandung City, Indonesia (32) pharmacies were used as distribution centers for the subsequent distribution of vaccines to remote areas. The above improved the vaccination coverage.

Overall, researchers agree that, to implement better PBI strategies, it is mandatory to design and develop adequately key aspects including the management of facilities, the management of patient records and patients' engagement (33), remuneration methods, the training of professionals, as well as the design of operating procedures (34). As the use of PBI strategies grows, various models of PBI have been elaborated to address these issues in different manners, depending on the specific situation and needs of each country. Table 1 summarizes their main characteristics, reporting for each case the type of professionals involved, how the services are funded, and the requirements that regulates the service.

**Table 1:** PBI strategy frameworks.

**Country**	**Vaccination, testing, and/or prescription provider**	**Payment**	**Record keeping**	**Regulatory requirements**
**PBI strategy frameworks**				
United States	Community pharmacists and pre-registration pharmacy students	Private and public health insurance	Electronic systems to have access to centralized public health records and avoid duplicating vaccine application	The pharmacist must be certified to prescribe, perform a test or administer a vaccine, vaccination without prescription can be done if it is part of an immunization program. The pharmacist must check in the database, if available, the patients' record to avoid mistakes.
Portugal	Doctor, community pharmacist	Government funded	Electronically	
England	Patient Group Direction (PGD) in community pharmacy or pharmacist	National Health Service for at risk groups and private payment for regular patients	Web-based software or paper-based data	
Canada	Pharmacists	Remuneration is provided by provincial/territorial government for vaccine administration at pharmacies. Patients can pay for vaccines injection fees at pharmacies if they require one	Electronic & cellphone application	

Retrieved from Kirkdale et al. (34), Federation et al. (96), and Fonseca et al. (97).

### 3.5. Benefits

Community pharmacies have been proved to have specific advantageous attributes to meet certain health demands and to increase patients' access to healthcare (35). Several advantages directly concern patients. First, pharmacies offer alternative and accessible points of care into complicated networks of healthcare providers (5, 27). Furthermore, immunization is safer at pharmacies than at other healthcare facilities, such as hospitals, where the risk of secondary infections associated with a visit is larger. Pharmacies offer a greater degree of territorial equity than other healthcare centers. This is particularly true for inhabitants of backward or difficult-to-access area (36, 37), for whom PBI improves accessibility, thus decreasing disparities (38–40). Second, a better accessibility translates into increased vaccination rates (20, 34), particularly in medically underserved populations (34, 38) and in adults (19, 41). Third, pharmacies have adequate staff to vaccinate and to answer patients' basic medical needs (5). Moreover, the contribution of pharmacists, nurses, and interns to immunization alleviates the workload of physicians. Finally, PBI improves the care level offered to patients and the involvement and enthusiasm to enhance the services (19), resulting in higher patients' satisfaction (27).

PBI also brings benefits for pharmacies and the healthcare system (38). Moreover, PBI brings profitable business opportunities for pharmacies and policy changes that expand pharmacy services, such as training for pharmacists and pharmacy staff on immunization practices. Several studies have reported improvements in both revenues (40) and cost-effectiveness of implementing PBI strategies for both, the patients, and the immunization facilities (27). Singhal and Zhang (42) claimed that the direct costs per adult influenza and pneumococcal vaccination are lower in pharmacies by 16–26% than in physician offices and other medical settings (11–20%). In the study by Prosser et al. (43), this cost reduction was attributed to patients' decreased time spent waiting and lower operation costs. Furthermore, a study on community pharmacy-based immunization in the United States (44) found that most pharmacies had a positive annual net profit, thus demonstrating the potential for PBI to sustain well into the future.

Finally, pharmacies can contribute to the collection and update of patients' medical data such medications they take, medical history, immunization history, and location (27).

### 3.6. Challenges

First, although the increase in vaccination rates with PBI is highlighted, most studies that confirm this are based on surveys; therefore, it only represents the vaccination rates among a group of individuals under certain conditions and not those of the entire population and countries in general (45).

One of the main challenges in PBI is communication. PBI faces challenges such as the lack of patients' knowledge and confidence of the training, competences, and abilities that immunizers have (46), the current structure of immunization delivery in the community, and the volume of immunizations to be administered (19). Furthermore, even though collaborations with insurers have been proposed, insurers still hesitate to pay and collaborate with pharmacies because of the lack of evidence that demonstrates the value and demand for the service (29).

In the US, other challenges have been identified regarding community pharmacies as immunization centers, including a limited number of commercial and government health plans that offer patients coverage to receive vaccines from a pharmacy, the lack of technology and patient registration systems, and the cost for the vaccine product itself and the cost for its administration. Another challenge is that, according to Inguva et al. (47), there are big disparities in the use of nontraditional vaccination settings by age and race or ethnicity; therefore, this problem should be examined before implementing PBI strategies. Bach and Goad (23) and Westrick and Breland (48) highlighted the effects of organization-level factors on the performance of PBI strategies and the effective execution of humanitarian supply chain strategies (49). Furthermore, political and organizational barriers limit the feasibility and effectiveness of the delivery of vaccines in pharmacies (38); therefore, uniformity in the laws and regulations is needed along the territories where a PBI strategy is implemented (50).

Because of the importance of multidisciplinary partnerships to increase access to healthcare services (28), the complexity of integration, participation, and coordination of multiple stakeholders, such as pharmacy schools, state and national pharmacy associations, pharmacy boards, state health departments, and federal agencies (51), should also be addressed in innovative ways. Therefore, conducting more theory-based studies is encouraged, focusing on pharmacy environmental factors and organizational characteristics, such as state regulations and immunization educational programs (51). Czech et al. (22) reported that legal and organizational frameworks must be developed to conduct vaccinations in pharmacies by pharmacists and to enhance the cooperation between all healthcare stakeholders. Finally, Fava et al. (52) underlined that the abundance of immunization programs can cause coordination problems between them and makes the identification of the most efficient and sustainable strategies in the different healthcare systems difficult.

The authors of these studies suggested future interventions in the correct training of personnel to provide vaccines efficiently and increase patient confidence (38), parallel to educating the population about the skills and reliability of personnel such as doctors or pharmacists in charge of administering the vaccine, Ernst et al. (19), Crawford et al. (53), and Calo et al. (54). Moreover they claimed that organizational challenges must be addressed at multiple levels for the implementation, planning, and logistics of PBI strategies to increase its compatibility with pharmacies that coincide with heterogeneous practice sites.

According to Guayta-Escolies et al. (5) and Fitzgerald et al. (55), PBI strategies are unique to each country's healthcare system, and although following standardized models applied in countries with more experience is useful, these models should be redesigned, and new flexible models, approaches, and designs that work under scenarios of limited economic resources, rationalization, polarization of services, and more effective public-private partnerships must be created. Therefore, PBI strategies must respond to vaccination needs based on local epidemiological data and should be coordinated with public health agencies for its correct operation.

The study by Sheffer et al. (56) emphasizes that key activities to improve community immunization include increasing immunization access points and hours (25), consistent patient education and communication, documenting results in patient medical records, monitoring quality measures, and integrating other patient care activities, such as diabetes mellitus control to better monitor risk and care. Consequently, the search for new strategies is necessary to achieve a proactive involvement (57) and collaboration between primary care physicians, public health officials, and pharmacists and better use of technology (34) whit access to electronic medical and health records for a better patients' information management. Klepser and Klepser (29) highlighted that pharmacists, prescribers, laboratorians, insurers, public health officials, and patients must collaborate to identify key point-of-care locations that are appropriate and sustainable in community pharmacies. Moreover, they recommended addressing pharmacogenetic testing to provide information on drug metabolism, sexually transmitted infections, and serum chemistries to support the optimization of drug therapies in the future.

In contrast, because in some countries, insurers cover vaccinations for some diseases, researchers recommend reviewing the bureaucratic processes between pharmacies, healthcare centers, and insurers to avoid bottlenecks, thus speeding up the vaccination process of the patients.

Finally (44) explained that external factors, such as competition between pharmacies from different chains, are a challenge to ensuring the sustainability of providing immunization services.

### 3.7. PBI strategies: Quantitative approaches

#### 3.7.1. Statistical approaches

The studies in this research labeled as “statistical” consisted of surveys, interviews, data collections, case studies, and experiments where statistical methods and analyzes were performed to obtain information, identify new patterns, and draw conclusions. Several statistical studies, such as the study by Westricka and Mountb (58), have been conducted to evaluate the behavior and effectiveness of the implementation of PBI strategies in different parts of the world.

***Location and accessibility**. *Magambo et al. (59) examined the relationship between the geographical location of healthcare facilities and the performance of immunization programs in Uganda. It was found that there is a deficiency in vaccination services in both rural and urban areas. The study attributes the poor vaccination performance to long waiting times in healthcare centers, which make patients prefer to forgo the service. Furthermore, the study suggested increasing the coverage by bringing immunization outreach services to rural residents at flexible hours. Van Amburgh et al. (60) conducted a demographic statistical analysis of a case study in the US where a PBI program was implemented in a rural community to evaluate its impact on the overall immunization rate. The program improved the influenza immunization access by 95% by mailing reminders that consisted of education packets for the population. Moreover, AlMahasis (61) highlighted the role of community pharmacies in implementing PBI strategies to improve the vaccination rates in rural areas. Milkman et al. (62) showed that one of the top-performing interventions corresponds to sending reminder messages to individuals to get vaccinated. Liao et al. (63) addressed the trends in elder's influenza vaccination rates and locations using data from the 2009 to 2017 Medicare Current Beneficiary Survey. It was found that PBI improved the vaccination rates among elder adults due to the accessibility benefit it offers. The study emphasized that vaccination in physician offices, clinics, and community pharmacies do not compete, but rather complement each other. A study conducted in El Paso, Texas, US, and Ciudad Juarez, Chihuahua, Mexico, indicated that considering the geographical characteristics, individuals are willing to scarify inconvenience in accessibility for affordability (low costs), availability (variety of providers), and accommodation (service hours) (64).

***Availability**. *Burson et al. (38) addressed the effects of the availability of vaccines in pharmacies for distribution to physicians and clinics, in a context where the pharmacies are vaccine suppliers as well. Different scenarios were examined, and regardless of the function that pharmacies have, as vaccine administrators or as suppliers, the study indicated that they contribute positively to public health, creating benefits, such as the improvement in vaccination rates in the community, the enhancement of the pharmacy practice, and the generation of additional revenues. Doucette et al. (65) suggested, as future research, addressing the pharmacy service availability in community pharmacies, including independent, chain, and mass merchandizers and supermarket pharmacies, to improve the capacity to deliver such services–immunizations, screening services, disease management programs, and medication therapy management services.

***Travel medicine**. *Some statistical studies have been directed toward travel medicine; Hind et al. (66) suggested that there is a great acceptance of the use of pharmacies as immunization centers among travelers who need specific vaccines, who, in addition, are willing to pay a fair amount for the service, which is less than what is paid when going to private healthcare centers. Nevertheless, further studies must be conducted to measure its cost-efficiency and rentability (30). The Pharmacy Partnership for Long-Term Care program was launched with pharmacy providers to manage the COVID-19 vaccination process among residents and staff members of long-term healthcare facilities. It increased the vaccination coverage and reduced workload for diverse entities by coordinating scheduling, vaccine cold chain management, patient counseling, and vaccine administration. In the first month after the implementation of the program, more than one million vaccines were administered (67).

#### 3.7.2. Mathematical optimization approaches

Several mathematical approaches have been proposed to address PBI-related problems, along with various solutions techniques, including hybrid simulation-optimization (27), queueing approximations for drive-through dispensing (28), and addressing demand uncertainty through chance constraints. Furthermore, among the models reviewed, some were developed specifically to solve PBI-related problems, and other models, although developed in immunization contexts where pharmacies are not explicitly considered, may be adapted for PBI needs.

***Location problems**. *Jia et al. (68) proposed a maximal covering model and three heuristics (a genetic algorithm heuristic, a locate-allocate heuristic, and Lagrangian relaxation heuristic) to determine the facility locations and allocation of medical supplies for large-scale emergencies with multiple facility under quantity-of-coverage and quality-of-coverage requirements. However, they recommended that if all demand must be satisfied at the same period, P-median or P-center formulations should be used rather than maximal covering. Singh et al. (69) presented an optimization approach to distribute antiviral drugs during the influenza pandemic in Texas, US. A pharmacy-based facility location problem was solved by designing a commercial pharmacy distribution network, which maximizes access by measuring the willingness-to-travel of individuals. This model resulted from a collaboration between academic researchers and public health officials and has been used by the Texas Department of State Health Services as a decision support tool.

After the COVID-19 pandemic, the US launched a program to maximize the immunization coverage among its inhabitants. This program consisted of creating alliances with pharmacy chains and the famous “dollar stores” to act as vaccination centers, taking advantage of their large distribution network throughout the country to reach marginalized communities. Bravo et al. (70) concluded that in this type of program, selecting the location of the pharmacies and stores that will be in charge is more critical than the quantity or capacity of these establishments. Therefore, Bravo et al. (70) showed a vaccination facility location model that decides which sites, among pharmacies and dollar stores, provide the immunization service and proposes a large-scale mixed-integer program that maximizes aggregate vaccination across all regions with demand coverage, budget, and capacity restrictions; however, these models do not seek 100% vaccination coverage. In the same study, the geospatial data showed that the inhabitants of marginalized areas were related to racism; therefore, this proposal (70) covered the location of healthcare facilities, rationing of vaccines, and equitable allocation of resources in healthcare. These efforts have improved both the equity and efficiency of vaccine distribution, and benefits have been observed in reduced travel distance (proximity to the population) and transportation costs to these sites.

Zhang et al. (71) presented an assignment model of antiviral drugs to urban pharmacies to identify the benefits of this distribution strategy in Shanghai. In this model, the selected group of pharmacies must meet multiple criteria related to access, social unbalance, and sparsely spatial distribution. An interesting conclusion drawn from this research is that the improvements in social and spatial unbalance were in the cost of access, which must be considered when implementing this strategy in real life.

In contrast, Risanger et al. (72) addressed the application of infection tests, a topic that although it is not focused on vaccination, addresses a key activity to contain the spread of infectious diseases. Risanger et al. (72) executed a willingness-to-travel estimate from the United States National Household Travel Survey data and recommended using pharmacies as COVID-19 testing centers in the US to improve the coverage/accessibility of the population to testing services. A facility location problem in conjunction with the willingness-to-travel estimate was solved, and the model maximized the population coverage considering a limited budget of locations, which can be selected using postcode mapping on the areas of interest. Some of the most outstanding aspects of the results obtained were: (1) densely populated states can simplify testing logistics by collaborating with a single pharmacy chain, whereas, (2) in states with a lower population density, working with more pharmacy chains is necessary to reach at least half of the population. Overall, this study showed that if the pharmacies with testing services were selected using this model in 1,000 ZIP code areas of the US, the service could be provided to 29 million more individuals than selecting the pharmacies based on population density alone.

***Economic approaches**. *Duncan et al. (73) addressed the reduction in economic losses because of a pandemic through a comprehensive actuarial model that can help private entities maximize their benefits through their immunization programs. This model incorporates an influenza season PBI strategy and can improve cost savings when targeting high-risks populations first and when the availability of pharmacies as vaccination centers increases. Furthermore, Fitzgerald (74) evaluated the cost-effectiveness of a PBI program for a high-risk influenza population in Nova Scotia, Canada, using a decision-tree model to estimate the effects of the pilot program on the population. The estimated savings were approximately $41,000, demonstrating the cost-effectiveness of PBI programs due to decreased hospitalizations and mortality. In the study, a retrospective analysis, surveys, an effectiveness model, and a model-based economic evaluation were used. Additionally, through an agent-based model and the simulation of different influenza scenarios, Bartsch et al. (75) have proven that the inclusion of pharmacies as vaccination centers increases cost-savings of third-party payers and the society.

### 3.8. Non-PBI: Quantitative approaches

Proposals based on vaccination strategies that do not explicitly consider pharmacies can be adapted and used to improve PBI strategies. These proposals consider facilities, such as small public healthcare clinics and distribution centers, as vaccination centers. Moreover, some of them consider the cold storage needed for the vaccines, and others deal with vaccination in remote locations. We first present studies related to the location of immunization facilities and then studies considering the entire vaccine supply chain. Finally, some innovative approaches, such as mobile clinics or drive-through settings, have been reported.

#### 3.8.1. Mathematical optimization approaches

***Location problems**. *Alghanmi et al. (76) compiled problems related to facility location for vaccines and drug distribution during health emergencies. These models might be easily adapted to PBI needs. Leithäuser et al. (77) used mathematical modeling techniques to optimally select the locations of vaccination centers among a given set, and to assign patients to the centers. The objective consisted of minimizing patients' travel distance and the number of required facilities and physicians. The study highlighted limitations in vaccine transportation, which requires special equipment to keep them at the right temperature, and affirmed that, unfortunately, public locations and local or small public health departments do not count with these types of resources. Lim et al. (78) proposed a robust mixed-integer programming model and a heuristic to redesign a vaccine distribution chain, which includes the location of intermediate distribution centers and determines the flows from the place where vaccines are received in a country to the healthcare clinics where vaccination occurs. The vehicles, the cold storage devices used at each clinic, and the characteristics of a country, such as population and size, are considered.

Bertsimas et al. (79) integrated a predictive SIR (Susceptible, Infected, Recovered) model into a prescriptive model to optimize the location of vaccination sites and subsequent vaccine allocation using a coordinate descent algorithm that iterates between optimizing vaccine distribution and simulating the dynamics of the pandemic. With this model, the effectiveness of the COVID-19 vaccination campaign in the US increased by approximately 20% by reducing the death toll of the pandemic in several states without hurting others and by achieving similar benefits under various perturbations in determining the locations of mass vaccination sites across the country and allocating vaccines among the population depending on age groups. Munguía-López and Ponce-Ortega (80) presented optimization strategies for the allocation of vaccines during the COVID-19 pandemic, and applied them in a case study in Mexico. These strategies aim to improve equity, and for the allocation of vaccines, they contemplate the size, risk profiles, and fraction of vulnerable groups in the population. In the case study, parameters such as the population, the case rate, the available beds, and the COVID-19 mortality rate of each state, were considered. This study showed that the complexity of assigning vaccines to each state increases with the availability of vaccines, because there are not always enough vaccines available to assign them to the entire population; therefore, fair allocation schemes must be developed.

Lusiantoro et al. (81) introduced a mathematical location-allocation model based on a maximal covering location problem to optimize the coverage of the COVID-19 vaccine distribution in a developing country, minimizing the risk of the virus transmission and transportation costs. Lim et al. (82) addressed the challenges to vaccinate low- and middle-income countries, adapting a facility location problem to four outreach coverage problems, where vaccines are taken to remote locations to maximize the population that can be reached. Along the same line of research, Goentzel et al. (83) developed a mixed integer program that determines the location of outreach sites and the resource deployment across healthcare centers and outreach sites, maximizing the immunization coverage within constrained budgets. This approach was applied to a case study from Gambia, and a 6.1% increment in the immunization coverage under the same budget was observed. Devi et al. (84) proposed a linear programming model to solve the location-allocation problem of temporary testing laboratories to detect influenza outbreaks. The model minimizes costs and the maximum traveling time of patients, and although it is not focused on vaccination, testing and vaccination happen under similar conditions. Everett et al. (85) highlighted that the employment of geospatial analytical methods to locate potential vaccination centers allows healthcare departments to adjust staffing, shifts, and the number of locations. Finally, Verma and Dash (86) used spatial coverage modeling to increase the coverage of healthcare services, including immunization, in rural, remote, and fragile areas of India.

***Vaccination supply chain**. *Sadjadi et al. (87) proposed a robust counterpart model to design an entire multi-echelon network of a vaccine supply chain considering the locations of the manufacturer, primary warehouses, and healthcare facilities and the perishability of vaccines, wastage in storage, limited capacity, different priorities for demands, and uncertainty.

Childhood vaccination programs are one of the most effective strategies to prevent deaths from infectious diseases and are considered to be one of the most cost-effective investments in terms of health. Paradoxically, infectious diseases are one of the main causes of mortality in low- and middle-income countries. Because of the aforementioned facts, some researchers have focused on studying vaccination in these countries, where vaccines are usually distributed *via* a hierarchical legacy medical network determined by political boundaries and history, where rigid structures predominate and are replicated in most of these countries. Therefore, Yang and Rajgopal (88) have suggested a mathematical programming model and a heuristic to minimize costs while improving the universal coverage in vaccine distribution networks for low- and middle-income countries avoiding sophisticated systems. The model considered the operational simplicity needed to execute it in different contexts.

Finally, Srivastava et al. (89) used a greedy adding algorithm-based optimization with healthcare facility-level geolocation data to straighten the immunization supply chain in eight districts of Madhya Pradesh, India.

#### 3.8.2. Innovative approaches

***The mobile clinic**. *Çakir et al. (90) examined a multifacility location problem formulation to locate mobile vaccination clinics so that the total costs are minimized in candidate areas of Istanbul, Ankara, and Izmir, Turkey, and proposed a Lagrange relaxation and a fuzzy saving heuristic to solve instances originating from the COVID-19 vaccination strategies in these regions. Hodgson et al. (91) used a covering tour model to locate the bases of mobile healthcare facilities to minimize mobile facility's travel and serve most of the population centers; they emphasized the importance of flexibility in the mobile systems to provide the service along the year. Similarly, Fadaki et al. (92) proposed a multiperiod vaccine allocation model using a capacity-sharing mechanism with mobile units.

***Drive-through facilities**. *Drive-through facilities have been used during the COVID-19 pandemic for testing and immunization activities. Asgary et al. (93) used a simulation tool integrating discrete events and agent-based modeling techniques for planning and designing mass vaccination facilities. This strategy is suitable for difficult-access communities and helps reduce contacts between healthcare workers and the population to be immunized.

Tables 2, 3, 4 in Appendix summarize the qualitative, statistical, and optimization contributions analyzed in this literature review.

## 4. Discussions and future research directions

Mass immunization and, more generally, the fight against infectious diseases have always been of paramount importance, particularly for the COVID-19 pandemic. Although immunization has been traditionally executed by family physicians, the needs of today's world point toward more flexible healthcare systems that adapt to the patients' needs, provide equal access, and reduce patients lost in the healthcare system. For instance, Ernst et al. (94) concluded that patients living in smaller towns are more likely to receive vaccinations in nontraditional settings, such as community healthcare departments, school nurses, and pharmacies, confirming the disparities in vaccination rates between race, place of residence, and employment status (95). It is therefore important to try and evaluate how PBI can help cope with these emerging needs and overcome the limits of traditional immunization.

This systematic literature review produced a final set of 85 relevant studies that were classified and then analyzed. Considering that immunization is a key topic in scientific research, we deem the number of works related to the design and organization of vaccination networks very limited. Nevertheless, the next paragraphs attempt to answer the proposed research questions using the information found in the collected studies and their analysis.

1. *RQ1. What have been the results of implementing PBI strategies?* All reported studies agreed on the fact that PBI is a good and effective strategy which contributes significant benefits (5, 19, 20, 27, 34–38, 38–44, 70, 71, 73, 79). These studies observed a greater accessibility to vaccination services, improved equity on the access to services due to the geographical distribution of pharmacies over the territory, higher vaccination rates in rural areas and among adult population, higher vaccination coverage, the reduction in workload in bigger healthcare entities, greater revenues for the pharmacies, reduction in vaccine waste, enhanced data gathering on patients living in backward areas, and some studies also showed the feasibility to provide additional services to vaccination in pharmacies, such as screening services and disease management programs. However, because studies on PBI implementation are limited to a few countries (see RQ2), the fact that its effectiveness may vary depending on the economic, political, and/or social situations of the place of its execution should be considered.
2. *RQ2. Where and how PBI has been executed?* According to the scientific publications found, the implementation of PBI is limited mostly to first-world countries (the US and Europe), although in practice other countries, such as Canada, have been executing vaccination in pharmacies for years. Furthermore, other sources reported PBI's implementation in other countries, such as Australia and India.

In the specific case of the COVID-19 pandemic, several governments bought vaccines (US, European countries, and Canada) and pharmacies contributed providing the immunization service to the population. These countries have a large and well-established network of pharmacy chains and community pharmacies that already contribute to the distribution of drugs in collaboration with insurance companies and public healthcare organizations. We believe that these previous alliances not related to immunization are key to the success of PBI strategies. One last factor facilitating the implementation of PBI in these countries is that the education of pharmacists already includes the certification with the necessary credentials to vaccinate. In short, it appears that the level of collaboration and trust required between parties in the implementation of PBI cannot be improvised and require previous alliances to succeed.

Furthermore, the lack of the implementation of PBI strategies in developing countries may be caused by the economic limitations that can be assumed for its implementation; for example, to collaborate in immunization campaigns with pharmacies, the governments and municipalities of these regions must have and allocate sufficient financial resources to subsidize part of the vaccines or their entirety to provide them completely free of charge and pay the pharmacies for the services they provide. However, although at first it may seem expensive, using effective immunization strategies will always be cheaper than saturating more insufficient public services with these activities. Hence, the importance of conducting scientific studies and case studies that prove it has been emphasized.

3. *RQ3. What should be necessary (from research) to support a better implementation of PBI strategies?* To better support the implementation of PBI, addressing planning and operational problems is necessary, including but not limited to the location of pharmacies, the management of the cold supply chain, the education of healthcare personnel and the population, the design of collaboration frameworks between the public sector (government and healthcare departments) and the private sector (various pharmacy chains), and their organizational structure in each country to explore the needs of the health sector and defining service limitations, such as whether vaccination is free or can be covered by some type of health insurance. Moreover, although some Latin American countries implement PBI strategies, no authors have documented their experiences or formally proposed an operation plan. The aforementioned issue leaves uncertainty in the reasons why there is no research in this area, not only for Latin America but also for the rest of the countries that have a good infrastructure and/or territorial distribution of pharmacies. The foregoing opens new paths to explore, such as limitations at the economic, political, and social levels of each country and the interaction between different public and private bodies. Furthermore, to implement these strategies, new rules and regulations must be developed for their proper management.

To summarize, our review confirmed the idea that PBI is already playing an important role in countries with large pharmacy networks in partnerships with public healthcare organizations. This role will even grow in the forthcoming years to complete and, in other cases, to improve the access of population to immunization services. In countries where primary healthcare services must develop, we believe that public health authorities should consider and integrate pharmacies as partner facilities as early as possible in their plans to provide primary healthcare services, including but not limited to immunization services. To this end, operations research tools, such as simulations, and various optimization models, including covering, location, districting, and resources allocation, should be used to design hybrid multi-service multi-provider networks to reinforce the offer and access to healthcare.

In this vein, this review has highlighted a lack of studies devoted to the development of optimization models and decision support tools for designing networks supporting PBI, which are, as it was previously mentioned, essential for an effective and efficient network design, allowing us to exploit the potential of PBI. Indeed, most available studies on PBI focused on its performance and on the acceptability between society and the health sector, suggesting that PBI has yet to demonstrate its potential and gain credibility from public decision-makers' perspective. Fortunately, the results reported in recent case studies and the successful contribution of pharmacies in the administration of vaccines during several waves of COVID-19 opened the door to new, more collaborative relationships between public health systems and pharmacies in terms of vaccination.

### 4.1. Future research directions

Additional research is required to clarify the terms and extent of that collaboration. For instance, although most studies agreed on the benefits of PBI, only 10% of them focused on rural, hard-to-reach areas, for which accessibility is key to receiving the services they need. We believe that studies targeting specifically that kind of areas are necessary to corroborate the expected suitability of PBI to cover their needs.

Furthermore, we believe that research efforts must be put to identifying and better understanding the barriers that slow down or inhibit the deployment of PBI in countries having developing or weak healthcare structure. In fact, although Latin American countries, such as Argentina, Brazil, Chile, and Colombia, have traditionally offered vaccination services in pharmacies, no scientific articles were found that report PBI experiences. We urge researchers to document these cases and develop customized models fitting the particularities and needs of each country or region. It is worth noting that, contrary to what one might think, the success of PBI is not only related to the number of pharmacies in each country. Indeed, according to Kirkdale et al. (20), the number of pharmacies per 10,000 inhabitants in the US is rather the same as that in Mexico and almost half of the one observed in Brazil or Paraguay.

Finally, as we already mentioned, our research has also confirmed the scarcity of studies contributing mathematical models to the design and management of PBI networks. However, we believe that immunization is not the foremost mission of pharmacies and that pharmacies' locations are given and set according to the profit-related criteria, although most likely including the proximity to the population, making the location problem less appealing to researchers. Furthermore, most successful case studies, if not all of them, focused on chains of pharmacies rather than independent pharmacies. The notorious lack of data on the healthcare system makes the validation of models, particularly quantitative mathematical models, difficult, which does not encourage its application in these types of problems. On a positive note, other mathematical models, although not focused on pharmacies, can be used in formulating PBI strategies with little or no adaptation.

## 5. Conclusion

This study reviewed the literature related to PBI and, more generally, the role of pharmacies alone or in collaboration with other public or private immunization facilities in mass vaccination networks. Our systematic literature review produced a final set of 85 relevant studies that were classified and then analyzed to answer the research questions motivating this work.

Our work contributes three main conclusions that suggest, simultaneously, relevant research directions. First, the scientific literature agrees on the implementation of PBI strategies in more countries because of its benefits–in a shell, better accessibility to vaccination services, territorial equity, and an increase in revenues for pharmacies–although additional and focused research should be conducted to contextualize them to specific situations, such as rural regions or hard-to-reach areas for which accessibility is key to receiving the services they need. Second, most case studies reported in the literature focused on developed countries, whereas our intuition suggests that the biggest potential of PBI should be revealed in developing countries with fragile public healthcare systems and infrastructure.

Third, documenting the experiences with PBI strategies is essential so that they may serve as a watershed for their implementation in other places with similar political and social conditions. Promoting PBI strategies requires the development of models and decision support tools to address challenging problems related to the design of networks, the management of the cold supply chain, and the collaboration between the public (government and healthcare departments) and private (various pharmacy chains) sectors and their organizational structures.

Furthermore, even if we agree that the use of PBI strategies increases accessibility to vaccination services and therefore improves the vaccination coverage, we also think that the combination of PBI with other strategies, such as mass vaccination campaigns or outreach strategies, the full vaccination coverage can be aspired to, regardless of individuals' desire to be vaccinated.

Finally, this study is also subject to some limitations. Although the literature review make us infer that the implementation of PBI strategies is a good idea, there is a possibility of not having access to unpublished initiatives that can have different results from those found. Moreover, the possibility of PBI application in more countries cannot be generalized because there is no access to commercial agreements between pharmacies, organizations, and public healthcare bodies.

## Data availability statement

The original contributions presented in the study are included in the article/Supplementary material, further inquiries can be directed to the corresponding author.

## Author contributions

MR-M, JM-V, and AR contributed to conception and design of the study. MR-M organized the database, performed the analysis of the results, and wrote the first draft of the manuscript. JM-V and AR commented on previous versions of the manuscript and AR performed final changes. All authors contributed to manuscript revision, read, and approved the submitted version.
