# Supplementary material for: Pharmacy-based immunization: a systematic review
Source: Front Public Health. 2023 Apr 13;11:1152556. doi: 10.3389/fpubh.2023.1152556 (PMC10133503; doi:10.3389/fpubh.2023.1152556)
Supplement: Supplementary file 1 [file Table_1.pdf]

1 APPENDIX

Tables 2 3 and 4.

Table 2. Literature review: qualitative studies

Autor	Year	Title	Journal	Country	Methods	Disease
Jackson, A.B., Humphries, T.L., et al. Jackson et al. (2004)	2004	Clinical Pharmacy Travel Medicine Services: A New Frontier	Annals of Pharmacotherapy	USA	Review	travel vaccines
Evenson, C.J., Gierach, M.R., et al. Evenson et al. (2005)	2005	Nursing Program Partners With Community to Implement Neighborhood Influenza Immunization Clinics	Home Health Care Management & Practice	USA	Review	influenza
Koonin, L.M., et al. Koonin et al. (2011)	2011	CDC's 2009 H1N1 vaccine pharmacy initiative in the United States: implications for future public health and pharmacy collaborations for emergency response	Disaster medicine and public health preparedness	USA	Report	influenza
Keimer, J.C. and Mousa, S.A. Keimer and Mousa (2012)	2012	Expanding the role of community pharmacy: an evaluation of generic drug discount programs, pharmacy based immunization services and convenient care clinics	Pharma Scholars Library	USA	Review	-
Sheffer, J. Sheffer (2013)	2013	Pharmacy-based immunizations: Getting the word out	Pharmacy Today	USA	Summary of a presentation	-
Bach, Albert and Goad, Jeffery Bach and Goad (2015)	2015	The role of community pharmacy-based vaccination in the USA: current practice and future directions	Integrated Pharmacy Research and Practice	USA	Review	influenza
Miller, R. and Goodman, C.Miller and Goodman (2016)	2016	Performance of retail pharmacies in low- and middle-income Asian settings: a systematic review	Health Policy and Planning	UK	Review, case study	-
Burson, R.C., Buttenheim, A.M., et al. Burson et al. (2016)	2016	Community pharmacies as sites of adult vaccination: A systematic review	Human Vaccines & Immunotherapeutics	USA	Review	influenza
Kirkdale, C.L., Nebout, G., et al. Kirkdale et al. (2016)	2016	Benefits of pharmacist-led flu vaccination services in community pharmacy Vaccination antigrippale parle pharmacien en officine: quel apport?	Annales Pharmaceutiques Françaises	France	Review	influenza
Kirkdale, C.L., Nebout, G., et al. Kirkdale et al. (2017)	2017	Implementation of flu vaccination in community pharmacies: Understanding the barriers and enablers	Annales Pharmaceutiques Françaises	France	Review	influenza
Fava, J.P., Colleran, J., et al. Fava et al. (2017)	2017	Adolescent human papillomavirus vaccination in the United States: Opportunities for integrating pharmacies into the immunization neighborhood	Human Vaccines & Immunotherapeutics	USA	Review	HPV
Klepser, D.G. and Klepser, M.E. Klepser and Klepser (2018)	2018	Point-of-care testing in the pharmacy: how is the field evolving?	Expert Review of Molecular Diagnostics	USA	Review	-
Guayta-Escolies, R., Urbiztondo, L., et al. Guayta-Escolies et al. (2018)	2018	Vaccination coverage and community pharmacy: A strategically necessary and operationally feasible binomial?	Vacunas	Spain	Review	influenza
Pattin, A. and Sherman, L. Pattin and Sherman (2018)	2018	Experiences Among African American Community Members With Pharmacy-Based Immunization Services in Detroit, Michigan	Journal of Pharmacy Technology	USA	Focus group	-
William A. Calo, Parth D. Shah, et al. Calo et al. (2019)	2019	Implementing pharmacy-located HPV vaccination: findings from pilot projects in five U.S. states	Human Vaccines & Immunotherapeutics	USA	Pilot project, case study	HPV
Adunlin, George and Murphy, Pilar et al. Adunlin et al. (2020)	2020	COVID-19: How Can Rural Community Pharmacies Respond to the Outbreak?	The Journal of Rural Health	USA	Review	COVID-19
Al Aloola, N., et al. Al Aloola et al. (2020)	2020	Community needs and preferences for community pharmacy immunization services	Vaccine	Saudi Arabia	Interview	-
Czech, M., Balcerzak, M., et al. Czech et al. (2020)	2020	Flu Vaccinations in Pharmacies—A Review of Pharmacists Fighting Pandemics and Infectious Diseases	International Journal of Environmental Research and Public Health	Poland	Review	influenza, COVID-19
Alsaleh, N. LePan (2020)	2020	Pharmacist-led flu vaccination services in community pharmacy: Experiences and Benefit	SPER Publications and Solutions Pvt. Ldt.	Saudi Arabia	Review	influenza
Eisenstadt, M., Ramachandran, M., et al.Eisenstadt et al. (2020)	2020	"COVID-19 Antibody Test/Vaccination Certification: There's an App for That,"	IEEE Open Journal of Engineering in Medicine and Biology	UK	Project development: prototype	COVID-19
Daniel, Casey L., Lawson, Frances et al. Daniel et al. (2021)	2021	Enrolling a rural community pharmacy as a Vaccines for Children provider to increase HPV vaccination: a feasibility study	BMC Public Health	USA	Interview, data, case study	HPV
Alghanmi, N., Alotaibi, R. et al. Alghanmi et al. (2022)	2022	A Survey of Location-Allocation of Points of Dispensing During Public Health Emergencies	Frontiers in Public Health	Saudi Arabia	Review	COVID-19
Kim, C., et al. Kim et al. (2022)	2022	COVID-19 Vaccine Provider Access and Vaccination Coverage Among Children Aged 5-11 Years - United States, November 2021-January 2022	MMWR. Morbidity and mortality weekly report	USA	Report	COVID-19
Kulczycki, A. and Shewchuk, R. Kulczycki and Shewchuk (2022)	2022	Back to Basics: A general approach to improving Covid and adult immunization delivery focused on Pharmacy-Based immunization services	Vaccine	USA	Review, strategy proposal	COVID-19
Lampasona, M. and Pantaleo L.Lampasona and Pantaleo (2022)	2022	The Role of Pharmacies in Immunization Programs and Health Promotion	Archives of Pharmacy Practice	Italy	Review	COVID-19

Table 3. Literature review: statistical approaches

Autor	Year	Title	Journal	Country	Methods	Disease
Ernst, M.E., Chalstrom, C.V., et al. Ernst et al. (1997)	1997	Implementation of a Community Pharmacy-Based Influenza Vaccination Program	Journal of the American Pharmaceutical Association	USA	Case study, data collection	influenza
Ernst, M.E., Bergus, G.R., and Sorofman, B.A. Ernst et al. (2001)	2001	Research: Patients' Acceptance of Traditional and Nontraditional Immunization Providers	Journal of the American Pharmaceutical Association	USA	survey	-
Van Amburgh, J.A., Waite, N.M., et al. Van Amburgh et al. (2001)	2001	Improved Influenza Vaccination Rates in a Rural Population as a Result of a Pharmacist-Managed Immunization Campaign	Pharmacotherapy	USA	Chart review	influenza
Van Amburgh, J.A., Waite, N.M., et al. Van Amburgh et al. (2001)	2001	Improved Influenza Vaccination Rates in a Rural Population as a Result of a Pharmacist-Managed Immunization Campaign	Pharmacotherapy	USA	Experiment	influenza
Doucette, W.R., Kreling, D.H., et al. Doucette et al. (2006)	2006	Evaluation of Community Pharmacy Service Mix: Evidence from the 2004 National Pharmacist Workforce Study.	Journal of the American Pharmacists Association	USA	Cross-sectional study	-
Goode, JV., Mott, D., and Stanley, DGoode et al. (2007)	2007	Assessment of an immunization program in a supermarket chain pharmacy	Journal of the American Pharmacists Association	USA	statistical analysis	influenza, pneumococcal vaccines, hepatitis A, hepatitis B, measles, mumps, rubella, varicella, meningococcal, and tetanus–diphtheria
Hind, C.A., Bond, C.M., et al. Hind et al. (2008)	2008	Needs Assessment Study for Community Pharmacy Travel Medicine Services.	Medicine Services. Journal of Travel Medicine	UK	questionnaire survey	travel vaccines
Prosser, L.A., O'Brien, M.A., et al. Prosser et al. (2008)	2008	Non-Traditional Settings for Influenza Vaccination of Adults	PharmacoEconomics	USA	Telephone interview	influenza
Usami, T., Hashiguchi, M., et al. USAMI et al. (2009)	2009	Impact of Community Pharmacists Advocating Immunization on Influenza Vaccination Rates among the Elderly	Yakugaku Zasshi	Japan	Experiment carried out, cluster randomized trial	influenza
Westrick, S.C., Watcharadamrongkun, S., et al. Westrick et al. (2009)	2009	Community pharmacy involvement in vaccine distribution and administration	Vaccine	USA	Survey	diphtheria–tetanus–pertussis, influenza, pneumococcal polysaccharide, travel vaccines
Westricka, S. and Mountb, J. Westricka and Mountb (2009)	2009	Impact of perceived innovation characteristics on adoption of pharmacy-based in-house immunization services	International Journal of Pharmacy Practice	USA	survey	-
Westrick S.C. and Breland M.L. Westrick and Breland (2009)	2009	Sustainability of pharmacy-based innovations: The case of in-house immunization services	Journal of the American Pharmacists Association	USA	cross-sectional study	-
Hess, K.M., Dai, C.-W., et al. Hess et al. (2010)	2010	Measuring outcomes of a pharmacist-run travel health clinic located in an independent community pharmacy	Journal of the American Pharmacists Association	USA	Survey	-
Crawford, N.D., Blaney, S., et al. Crawford et al. (2011)	2011	Individual- and Neighborhood-Level Characteristics Associated with Support of In-Pharmacy Vaccination among ESAP-Registered Pharmacies: Pharmacists' Role in Reducing Racial/Ethnic Disparities in Influenza Vaccinations in New York City	Journal of Urban Health	USA	statistical analysis	HIV
AlMahasis, S AlMahasis (2011)	2011	Impact of the COVID-19 Pandemic on Pharmacy-based Immunization in Rural USA: a crosssectional study of community pharmacists from five Southeastern States	Auburn University	USA	survey	influenza
Doucette, W.R., McDonough, R.P., et al. Doucette et al. (2012)	2012	Three-year financial analysis of pharmacy services at an independent community pharmacy	Journal of the American Pharmacists Association	USA	Examination of records	influenza, herpes zoster, pneumococcal immunization, cholesterol screenings, adherence management services
Goad, J.A., Taitel, M.S., et al. Goad et al. (2012)	2012	Vaccinations Administered During Off-Clinic Hours at a National Community Pharmacy: Implications for Increasing Patient Access and Convenience	The Annals of Family Medicine	USA	data bases , case study	influenza
Murphy, P.A., Frazee, S.G., et al Murphy et al. (2012)	2012	Pharmacy provision of influenza vaccinations in medically underserved communities.	Journal of the American Pharmacists Association	USA	retrospective study, statistical analysis	influenza
Boda, P. J. Boda (2013)	2013	Medications in Mexico: The Growth and Distribution of Pharmacies in Ciudad Juárez, Chihuahua, Mexico from 1996-2011	International Journal of Geosciences	USA	cross-sectional questionnaire survey	-
Singhal, P.K., and Zhang, D. Singhal and Zhang (2014)	2014	Costs of adult vaccination in medical settings and pharmacies	Journal of Managed Care Pharmacy	USA	data bases	herpes, pneumococcal vaccine, influenza
Marra, F., Kaczorowski, J., et al. Marra et al. (2014)	2014	Pharmacy-based Immunization in Rural Communities Strategy (PhICS): A community cluster-randomized trial	Canadian Pharmacists Journal	Canada	cluster-randomized trial	influenza
Nouvellet, C. and Sauvant-Rochat, M.P. Nouvellet and Sauvant-Rochat (2015)	2015	Pharmacy-based immunization in France: visions of pharmacist, of physician and of patient: Marie-Pierre Sauvant-Rochat	European Journal of Public Health	France	cross-sectional study	-
Maceira,D., Goguadze, K., and Gotsadze, G. Maceira et al. (2015)	2015	The drivers of facility-based immunization performance and costs. An application to Moldova	Vaccine	Moldova	least squares regression analysis	-
McConeghy, K.W. McConeghy (2015)	2015	The Impact of Pharmacy-Based Immunization on Vaccination Rates and Prevalence of Influenza Virus	Thesis	USA	survey	influenza
Fitzgerald, T.J., Kang, Y. et al. Fitzgerald et al. (2016)	2016	Integrating pharmacies into public health program planning for pandemic influenza vaccine response	Vaccine	USA	case study, data analysis, experiment	influenza
McConeghy, K.W., and Wing, C. McConeghy and Wing (2016)	2016	A national examination of pharmacy-based immunization statutes and their association with influenza vaccinations and preventive health	Vaccine	USA	experiment	influenza
Inguva, S., Sautter, J.M., et al. Inguva et al. (2017)	2017	Population characteristics associated with pharmacy-based influenza vaccination in United States survey data	Journal of the American Pharmacists Association	USA	statistical analysis, regression	influenza
Islam, J.Y., Gruber, J.F. et al. Islam et al. (2017)	2017	Opportunities and Challenges of Adolescent and Adult Vaccination Administration Within Pharmacies in the United States	Biomedical Informatics Insights	USA	statistical analysis, interview	influenza, pneumococcal, TDAP, MCV4, HPV
Sokolow, L.Z., et al. Sokolow et al. (2018)	2018	Scripted Surge Pharmacy Pandemic Exercise: Testing Vaccine Administration and Antiviral Dispensing	Health security	USA	Case study, simulaton	influenza
Westrick, S.C. et al. Westrick et al. (2018)	2018	National survey of pharmacy-based immunization services	Vaccine	USA	survey	influenza, pneumococcal 13-valent conjugate, herpes zoster, pneumococcal polysaccharide, adolescent vaccinations
Magambo, N.K., Bajunirwe, F. and Bagenda, F. Magambo et al. (2020)	2020	Geographic location of health facility and immunization program performance in Hoima district, western Uganda: a health facility level assessment	BMC Public Health	Uganda	Interview, data, case study	-
Liao, Mott, D.A., Ford II, J.H., et al. Liao et al. (2021)	2021	Influenza vaccination rates and location for receiving the influenza vaccination among older adult Medicare beneficiaries	Journal of the American Pharmacists Association	USA	statistical analysis of data	influenza
Gharpure R., Guo A., et al. Gharpure et al. (2021)	2021	Early COVID-19 First-Dose Vaccination Coverage Among Residents and Staff Members of Skilled Nursing Facilities Participating in the Pharmacy Partnership for Long-Term Care Program — United States, December 2020–January 2021	Report MMWR Early Release	USA	statistical analysis	COVID-19
Olatunji, E.A., Ogunsola, A.S., et al. Olatunji et al. (2021)	2021	Who receives influenza vaccinations at the Pharmacy? An analysis of the Texas Behavioral Risk Factor Surveillance System	Vaccine	USA	statistical analysis	influenza
Murray, E., Bieniek, K., et al. Murray et al. (2021)	2021	Impact of pharmacy intervention on influenza vaccination acceptance: a systematic literature review and meta-analysis	International Journal of Clinical Pharmacy	France, USA	meta-analysis, statistical analysis	influenza
Milkman, K.L., Gandhi, L., et al. Milkman et al. (2022)	2022	A 680,000-person megastudy of nudges to encourage vaccination in pharmacies	Proceedings of the National Academy of Sciences	USA	statistical analysis, experiment	influenza

Table 4. Literature review: optimization approaches

Autor	Year	Title	Journal	Country	Methods	Disease	Vaccination	PB	Costs	FL
Hodgson, M. J., Laporte, G., and Semet, F. Hodgson et al. (2002)	2002	A Covering Tour Model for Planning Mobile Health Care Facilities in SuhumDistrict, Ghama	Journal of Regional Science	Ghana	Mobile-clinics, covering model	-				X
Fitzgerald, N. Fitzgerald (2006)	2006	Cost-effectiveness of pharmacy-based influenza immunization programs :a case study in the Capital District Health Authority, Halifax, Nova Scotia	-	Canada	Thesis	influenza			X	
Jia, H., Ordóñez, F. and Dessouky, M.M.Jia et al. (2007)	2007	Solution approaches for facility location of medical supplies for large-scale emergencies	Computers and Industrial Engineering	USA	Facility location, heuristic	-			X	X
Duncan, I.G., Taitel, M.S., et al. Duncan et al. (2012)	2012	Planning influenza vaccination programs: a cost benefit model	Cost Effectiveness and Resource Allocation	USA	Allocation problem, case study	influenza	X		X	
Everett, K.H., Potter, M.A. et al. Everett et al. (2013)	2013	Geospatial Analytics to Evaluate Point-of-Dispensing Sites for Mass Immunizations in Allegheny County, Pennsylvania	Journal of public health management and practice	USA	GIS, location-allocation algorithms, case study	-	X			X
Singh, B., Huang, H.-C., et al. Singh et al. (2015)	2015	Optimizing Distribution of Pandemic Influenza Antiviral Drugs	Emerging Infectious Diseases	USA	Facility location, case study	influenza				X
Lim, J., et al. Lim et al. (2016)	2016	Coverage models to determine outreach vaccination center locations in low and middle income countries	Operations Research for Health Care	India	case study, location-allocation	-	X			X
Bartsch, S.M., Taitel M.S. et al. Bartsch et al. (2018)	2018	Epidemiologic and economic impact of pharmacies as vaccination locations during an influenza epidemic	Vaccine	USA	agent-based model	-	X	X	X	
Zhang, C., Du, Z., et al. Zhang et al. (2019)	2019	Assignment optimization of pandemic influenza antiviral drugs in Urban pharmacies	Journal of Ambient Intelligence and Humanized Computing	China	Assignment problem, case study	influenza		X		
Sadjadi, S., et al. Sadjadi et al. (2019)	2019	The design of the vaccine supply network under uncertain condition: A robust mathematical programming approach	Journal of Modelling in Management	Iran	case study, location-allocation	-	X			X
Lim, J., et al Lim et al. (2019)	2019	Redesign of vaccine distribution networks	International Transactions in Operational Research	sub-Saharan Africa	case study, location-allocation, heuristics	-	X			X
Risanger, S., Singh, B., et al. Risanger et al. (2020)	2020	Selecting Pharmacies for COVID-19 Testing to Ensure Access	Health Care Management Science	USA	Facility location	COVID-19		X		X
Leithäuser, N., Schneider, J., et al. Leithäuser et al. (2020)	2020	Quantifying Covid19-vaccine location strategies for Germany	BMC Health Services Research	Germany	Heuristic, assignment problem	COVID-19				X
Verma VR and Dash U. Verma and Dash (2020)	2020	Geographical accessibility and spatial coverage modelling of public health care network in rural and remote India	PLoS ONE	India	GIS, coverage model, case study	-	X			X
Yang, Y. and Rajgopal, J. Yang and Rajgopal (2020)	2020	An Iterative Cyclic Algorithm for Designing Vaccine Distribution Networks in Low and Middle-Income Countries	Proceedings on 25th International Joint Conference on Industrial Engineering and Operations Management	USA	location-allocation, heuristics	-	X			X
Bertsimas, D., Digalakis Jr, V., et al. Bertsimas et al. (2022)	2021	Where to locate COVID-19 mass vaccination facilities?	Naval Research Logistics	USA	Facility location	COVID-19	X			X
Çakır, Taş, M. A. and Ulukan, Z. Çakır et al. (2021)	2021	Spherical bipolar fuzzy weighted multi-facility location modeling for mobile COVID-19 vaccination clinics	Journal of Intelligent & Fuzzy Systems	Turkey	Multi-facility location problem, heuristic	COVID-19	X		X	X
Munguía-López, A., and Ponce-Ortega, J. M. Munguía-López and Ponce-Ortega (2021)	2021	Fair Allocation of Potential COVID-19 Vaccines Using an Optimization-Based Strategy	Process Integration and Optimization for Sustainability	Mexico	Allocation scheme, case study	COVID-19	X			
Devi, Y., Patra, S. and Singh, S.P. Devi et al. (2021)	2021	A location-allocation model for influenza pandemic outbreaks: A case study in India	Operations Management Research	India	Facility location	influenza				X
Srivastava, V., et al. Srivastava et al. (2021)	2021	Strengthening the immunization supply chain: A time-to-supply based approach to cold chain network optimization & extension in Madhya Pradesh	Vaccine	India	case study, location-allocation, heuristics	-	X			X
Fadaki, M., Abareshi, A. et al. Fadaki et al. (2022)	2022	Multi-period vaccine allocation model in a pandemic: A case study of COVID-19 in Australia	Transportation Research Part E: Logistics and Transportation Review	Australia	case study, allocation model	COVID-19	X			X
Goentzel, J., et al. Goentzel et al. (2022)	2022	Vaccine network design to maximize immunization coverage	Journal of Humanitarian Logistics and Supply Chain Management	Gambia	case study,location-allocation	-	X			X
Bravo, F., Hu, J., and Long, E. Bravo et al. (2022)	2022	Optimal COVID-19 Vaccination Facility Location	SSRN	USA	Facility location, case study	COVID-19	X			X
Lusiantoro, L., et al. Lusiantoro et al. (2022)	2022	A Locational Analysis Model of the COVID-19 Vaccine Distribution	Operations and Supply Chain Management: An International Journal	Indonesia	case study, location-allocation	COVID-19	X			X

Table 4. Note. PB= Pharmacy-based, FL= Facility location
